# A phase III placebo-controlled study in advanced head and neck cancer using intratumoural cisplatin/epinephrine gel

**DOI:** 10.1038/sj.bjc.6600588

**Published:** 2002-10-21

**Authors:** J A Werner, W Kehrl, A Pluzanska, O Arndt, K M Lavery, J Glaholm, A Dietz, G Dyckhoff, S Maune, M E Stewart, E K Orenberg, R D Leavitt

**Affiliations:** Department of Otolaryngology, Philipps – University of Marburg, Deutschausstrasse 3, Marburg D-35037, Germany; ENT Department, University Hospital Eppendorf, Hamburg 20246, Germany; Department of Chemotherapy, Katedra Oncology Centre, Lodz 93-509, Poland; ENT Department of the Marienkrankenhaus, Regensburg 93053, Germany; Department of Oral and Maxillofacial Surgery, The Queen Victoria Hospital NHS Trust, West Sussex RH19 3DZ, UK; Birmingham Oncology Centre, Queen Elizabeth Hospital, Birmingham B15 2TH, UK; Department of Otorhinolaryngolgy, Head and Neck Surgery, University of Heidelberg, Heidelberg 69120, Germany; Department of Otorhinolaryngology, Head and Neck Surgery, University of Kiel, Kiel 24105, Germany; Department of Biostatistics, Matrix Pharmaceutical, Inc, Fremont, California, CA 94555, USA; Department of Professional Services, Matrix Pharmaceutical, Inc., Fremont, California, CA 94555, USA; Department of Medical Affairs, Matrix Pharmaceutical, Inc., Fremont California, CA 94555, USA

**Keywords:** head and neck squamous cell carcinoma, intratumoural injection, cisplatin, chemotherapy, randomised control trial, recurrence

## Abstract

Patients with recurrent or refractory head and neck squamous cell carcinoma received cisplatin/epinephrine injectable gel or placebo gel injected directly into the clinically dominant tumour. The double-blind phase III trial comprised of up to 6 weekly treatments over 8 weeks, 4 weekly evaluation visits, and then monthly follow-up; open-label dosing began as needed after three blinded treatments. Tumour response was defined as complete (100% regression) or partial (50–99% regression) sustained for ⩾28 day, and patient benefit as attainment of palliative or preventive goals prospectively selected by investigators and patients. With cisplatin/epinephrine gel, 25% (14 out of 57) of tumours responded (16% complete regression, 9% partial regression), *vs* 3% (one out of 35, complete regression) with placebo (*P*=0.007). Patient benefit was positively associated with target tumour response in the blinded period among cisplatin/epinephrine gel recipients (*P*=0.024): 43% (six out of 14) of responders benefited, *vs* 12% (five out of 43) of non-responders. The most frequent adverse event was pain during injection and the next most frequent was local cytotoxic effects consistent with the gel's mode of action. Systemic adverse events typical of intravenous cisplatin were uncommon. Intratumoural therapy with cisplatin/epinephrine gel provided safe, well-tolerated, effective palliative treatment for patients with locally advanced head and neck squamous cell carcinoma, who lack other satisfactory treatment options.

*British Journal of Cancer* (2002) **87**, 938–944. doi:10.1038/sj.bjc.6600588 www.bjcancer.com

© 2002 Cancer Research UK

## 

Despite aggressive early therapy, resistant head and neck squamouse-cell cancers (HNSCC) often recur, accompanied by distressing symptoms (Khuri *et al*, 2000). Options for local control in relapse are limited: prior extensive surgery and radiation therapy raise the risk of unacceptable complications with additional similar interventions. The intensification chemotherapy regimens required for effective local-regional control are highly toxic (Pignon *et al*, 2000), particularly in debilitated late-stage patients who typically develop recurrent head and neck cancer (Havlin *et al*, 1989; Robbins *et al*, 1994).

Intratumoural chemotherapy has been investigated for managing treatment-resistant tumours by exposing cancers to more cytotoxic agent than feasible with systemic dosing, while minimizing systemic exposure and adverse effects. Cisplatin/epinephrine injectable gel (CDDP/epi gel or IntraDose®; Matrix® Pharmaceutical, Inc, Fremont, CA, USA (Drug licensed to Chiron Corporation, Emeryville, CA, USA)) contains cisplatin and epinephrine in a biocompatible and biodegradable aqueous gel matrix of purified bovine collagen. The gel is injected directly into tumours under visual guidance or aided by cross-sectional imaging techniques. The gel's viscosity and epinephrine-induced vasoconstriction delay cisplatin clearance from tumours, prolonging exposure of malignant cells and sparing distant sensitive normal tissues (Mok *et al*, 2001). Intratumoural platinum concentrations 10 to 100 times those after systemic cisplatin treatment have been recorded for 1 to 3 days (Yu *et al*, 1995). In clinical experience with CDDP/epi gel in solid tumours, adverse effects typical of systemically administered cisplatin were rare and mild (Burris *et al*, 1998). Efficacy and clinical benefit of the drug for local tumour control has been demonstrated in phase II open-label trials for a variety of solid tumours including cutaneous and soft tissue metastases of malignant melanoma (Oratz *et al*, 2002), recurrent breast cancer (Roshon, 2001), oesophageal (Harbord *et al*, 2002), and gastric cancer (Monga *et al*, 1998), and primary and secondary malignant liver tumours (Leung *et al*, 2001; Thuluvath *et al*, 2001; Vogl *et al*, 2002).

The present study was a randomised, double-blind, placebo-controlled investigation of the safety and efficacy of CDDP/epi gel in patients with recurrent or refractory head and neck squamous cell carcinoma. We hypothesized that regression of the clinically dominant tumour consequent to local treatment with CDDP/epi gel would improve patient well-being by relieving or preventing the most life-impacting symptom. To explore this hypothesis, we evaluated the response of the clinically dominant tumour; Patient benefit, an endpoint based on the attainment of prospectively selected treatment goals; and the relationship between these two variables. The protocol and consent forms were approved by the Institutional Review Board at each study site before study initiation, all patients signed a written informed consent before enrollment, and the study was conducted in accordance with Good Clinical Practices.

## MATERIALS AND METHODS

### Eligibility and study entry

Entrants were required to have histologically confirmed, recurrent or refractory, primary or metastatic head and neck squamous cell carcinomas. Previous treatment with ⩾one course of therapy was obligatory, but patients must have recovered fully from any adverse effects of prior treatment. Patients who had a change in clinical status but required no intravenous chemotherapy were allowed to participate in the study. It was mandated that patients have tumours that were problematic and requiring intervention for the local problem. Tumours considered for treatment had to be 0.5 to ⩽2 cm^3^ in volume, readily measurable, accessible for direct intratumoural injection, and pose no immediate risk of haemorrhage or embolisation. Karnofsky Performance Status (KPS) of ⩾40, later amended to ⩾60, and an expected survival of at least 6 months were required.

Patients were excluded if they had New York Heart Association Class III or IV cardiovascular symptoms or a history of cardiac arrhythmia that might increase their risk of arrhythmia upon dosing; a tumour of the head or neck other than squamous cell carcinoma; or a history of clinically significant extracranial carotid vascular disease. Those hypersensitive to cisplatin, bovine collagen, epinephrine, or sulfites were excluded. Fibrotic or infected tumours were excluded from study treatment, as were tumours involving a major artery or visceral organ, directly involving or threatening to invade the carotid artery, or close to a major extracranial vessel.

Use of non-study cancer therapy or investigational agents was prohibited within 28 days before and throughout the trial. Concurrent use of drugs that interact with cisplatin was prohibited. Patients with no local tumour benefit could continue stable maintenance doses of hormonal therapy and radiation therapy to distant, non-target metastases that developed during the study. Bupivacaine HCl, cytotoxic or immunomodulating agents, and corticosteroids were prohibited during the trial (except stable steroid doses for chronic disease, asthma, or anti-emesis). Local or topical anaesthetic agents not containing epinephrine or adrenaline were permitted.

### Patient assignment and treatment

Investigators identified a target tumour that met the entry criteria and was the most symptomatically troublesome or threatening (e.g., to obstruct airway), and selected a palliative or preventive treatment goal for this tumour (other tumours could be treated but were not associated with the primary goal). Patients also selected a palliative goal. According to a stratified randomisation scheme (using a computerised list maintained by the sponsor), investigators assigned patients in a 2 : 1 ratio to treatment with CDDP/epi gel or placebo gel, within stratum I (target tumour volume <5 cm^3^) or stratum II (target tumour volume >5 but ⩽20 cm^3^). Cisplatin/epinephrine gel 1 ml contained cisplatin 4 mg, epinephrine 0.1 mg, purified bovine atelopeptide collagen matrix 20 mg, and excipients; placebo gel 1 ml contained purified bovine atelopeptide collagen matrix 20 mg and saline 0.9%. Doses were injected intratumourally at 0.25 ml cm^−3^ of treated tumour volume. Volume was estimated as length×width×height×0.5. Any portion of the 10-ml maximal permitted dose remaining after target tumour injection could be used to treat other qualifying tumours.

Before starting treatment, the investigator developed a pain management programme including, as needed, topical and local anaesthetics, local-regional nerve blocks, and systemic agents. Patients were treated as outpatients or during a brief hospital stay. Using a 22- to 30-gauge needle Luer-lock syringe, with a fanning or grid technique, the physician injected the gel in tracks about 1.0 cm apart throughout the entire tumour and a 1- to 2-cm zone surrounding the tumour margin. Extreme care was used to avoid injecting gel into a blood vessel. Investigators were instructed to inject the total gel volume slowly, in 2.5-ml increments, waiting about 5 min between injections, and checking pulse and blood pressure immediately and about 5 min after each incremental administration. Wound care measures included conservative wound management, early use of antibiotics for suspected infection, and delays in therapy if necessary.

Up to 6 weekly treatments were administered in an 8-week period (blinded treatment phase), followed by evaluations weekly for 4 weeks and then monthly for 5 months until study discontinuation or disease progression. If after three treatments the tumour(s) had progressed or only partially responded, patients could enter an open-label phase to receive active drug. Investigators, patients, and sponsor remained blinded to patients' initial treatment assignments until the last patient enrolled in the study completed 6 months of follow-up. Blinding was ensured by packaging CDDP/epi gel in identical cartons. At the study site, test drug was prepared by a pharmacist then given to the physician for administration.

### Evaluations

Volume of tumours to receive study treatment was measured at each visit. Response of the target and other treated tumours was based on maximal decrease from baseline in tumour volume during the blinded phase. Tumour response was classified as complete response (CR, 100% reduction in volume lasting ⩾28 days); partial response (PR, 50–99% reduction in volume lasting ⩾28 days); stable disease (⩽50 reduction in or ⩽25% increase in detectable, evaluable malignant disease; i.e., no significant measurable change); or progressive disease (>25% increase in detectable, evaluable malignant disease).

Rate of patient benefit was assessed based on attainment of prospectively chosen primary treatment goals defined in a new, validated instrument for measuring benefit associated with treating local tumours, the Treatment Goals Questionnaire^©^ (Werner *et al*, 2000; Mackowiak *et al*, 2001). Investigators selected an improvable primary treatment goal for the target tumour from eight palliative options (wound care; pain control; ability to see, hear, or smell; physical appearance; obstructive symptoms; and mobility) or three preventive options (invasion of vital structures and/or blood vessels, obstruction, or subcutaneous tumours breaking through the skin). Patients identified an improvable primary treatment goal for the target tumour from the eight palliative options. Attainment of palliative treatment goals was evaluated at screening, week 4, end of follow-up, and the last study visit, and attainment of preventive treatment goals at each treatment and follow-up visit. Patient benefit was achieved only if both patient and physician said the primary treatment goal was attained or one said the goal was attained and the other said that tumour-related signs and symptoms relevant to the goal did not worsen.

The active and placebo gels were also compared during the blinded phase for rate of response of all tumours treated; duration of and time to response of the target tumour and all tumours treated; time to progression of the target tumour and all tumours treated; and quality of life, as measured by the FACT-H&N (Cella, 1993) administered at weeks 1 and 4 and the end of follow-up.

Treated tumour sites were examined at each visit using a four-point scale (none, mild, moderate, severe) to assess symptoms of erosion, erythema, eschar, necrosis, swelling, and ulceration. Safety evaluations at each visit also included physical examination; blood pressure and pulse (pre- and post-treatment for treatment visits); laboratory evaluations (differential blood cell counts; haemoglobin and haematocrit levels; serum chemistries; serum pregnancy test, if appropriate, at screening); change in Karnofsky Performance Status (KPS); and adverse events (immediate injection effects, sustained local reactions, and systemic/other local reactions at a gel-treated site). Toxicity grading was based on NCCOG/NCI Toxicity Criteria (NCCOG/NCI, 1991).

### Statistical considerations

Planned enrollment was 90 patients (60 active-gel and 30 placebo-gel group recipients), the number required to provide a power of ⩾0.80 to detect a difference in response rates conditional on target tumour volume, using a two-tailed α level of 0.05 (Muñoz and Rosner, 1984). Primary efficacy analyses for the blinded treatment phase were comparison of the co-primary endpoints of target tumour response rates and patient benefit rates across treatment groups using the Cochran-Mantel-Haenszel (CMH) exact test for the overall analyses and the Fisher's exact test for analyses by stratum. Because co-primary endpoints were used, no adjustment for multiplicity was required. The association of target tumour response with patient benefit was also examined by the CMH exact test. Secondary analyses included change in FACT-H&N score and KPS, time to and duration of target tumour response, effect of covariates on target tumour response by logistic regression testing, and response of all treated tumours.

## RESULTS

### Patient disposition

Twenty-seven investigators enrolled patients at 28 centres in Europe and Israel, from June 1995 to March 2000. Patients included those who had received previous treatment with one or more modalities, had failed previous chemotherapy, were not candidates for further therapy, or refused therapy. Ninety-two patients (57 active, 35 placebo) were enrolled in the double-blind phase: 48 in stratum I (31 active, 17 placebo) and 44 in stratum II (26 active, 18 placebo). One hundred and eleven tumours were treated. In the blinded phase, of the 57 patients assigned to CDDP/epi gel, 25 (44%) completed the planned six treatments and 32 (56%) did not, including 3 (5%) who had an early CR, 3 (5%) whose systemic disease progressed, 15 (26%) whose target tumour progressed, 5 (9%) who had unacceptable adverse events, and 6 (11%) who withdrew for other reasons. Among the 35 patients assigned to placebo, 6 (17%) completed six treatments and 29 (83%) did not, including four (11%) whose systemic disease progressed, 18 (51%) whose target tumour progressed, and seven (20%) for other reasons (there were no unacceptable adverse events). Of the 29 placebo gel recipients who withdrew from treatment during the blinded phase, 24 (12 in each stratum) elected to cross over to the open-label phase to receive active drug.

### Demographics and baseline disease characteristics

The treatment groups were well matched for all demographic and baseline disease characteristics (Table 1

**Table 1:**
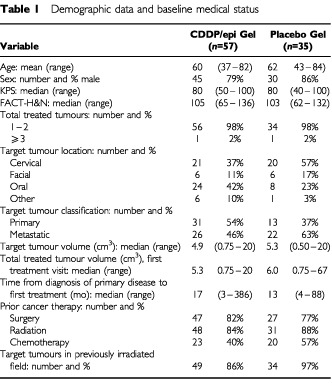
Demographic data and baseline medical status

). Baseline KPS was ⩽80 in 53 patients (58%) and ⩽70 in 13 (14%), indicating moderate function in most patients. One tumour was treated in 90% (83 out of 92) of patients at the first treatment visit; the greatest number of tumours treated in one patient was five. Patients had long-standing disease and were heavily pre-treated: 59% had a neck dissection; 66% had tracheotomy, glossectomy, and/or laryngectomy or laryngo-pharyngectomy; and 19% had received two or more cycles of chemotherapy. Most target tumours were in the neck and oral cavity, and 90% of them were in a previously irradiated field.

### Target tumour response

During the blinded phase, CR or PR was achieved in 14 patients (25%, Table 2

**Table 2:**
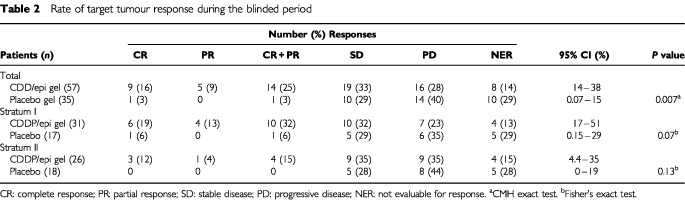
Rate of target tumour response during the blinded period

) treated with CDDP/epi gel; one placebo recipient had a CR (*P*=0.007, exact CMH). Duration of response after active gel therapy was a median of 64 days (stratum I, 61 days; stratum II, 228 days). Twelve of the 14 target tumours that responded to the active gel were still in local remission when the patients left the study. Median maximal tumour shrinkage in the five active gel recipients with PR was 94% (range, 60–99%). Among responders, the target tumour was in the oral cavity in 10, the neck in two and the face in two. Stratum I target tumours responded more often (32%) than stratum II target tumours (15%). The response rate was higher in patients who had no prior systemic chemotherapy (32%) than in those who did (13%), and lower in those who had prior platinum-based chemotherapy (11%) than in those who did not (32%), but these differences were not significant (*P*=0.086 and *P*=0.19, respectively, logistic regression analysis). Response of the target tumour to CDDP/epi gel became more likely as the number of treatments increased: 42% (10 out of 24) of target tumours responded with five–six treatments, 12% (three out of 25) with three–four treatments, and 13% (one out of eight) with one–two treatments.

Among 24 patients who failed placebo treatment and then received CDDP/epi gel, seven (21%) responded after a median of four (range, three–five) active gel treatments. Of patients who received active gel during either blinded or open-label phases, 21 out of 81 (26%) had target tumour responses (12 CR, 9 PR), with 15 out of 43 (35%) of the responders in stratum I and six out of 38 (16%) in stratum II.

Time to target tumour response to CDDP/epi gel in the blinded phase was a median of 53 days (range, 10–162); however, in many patients tissue conditions and tumour location delayed detection of response. Response to CDDP/epi gel occurred after a median of 4.5 treatments (range, two–six). Mean time to target tumour progression was 128 days (range, five–564) in the active group and 44 days (range, six–210+) in the placebo group.

### Patient benefit

Physicians most frequently selected wound care (25%) and pain control (24%) as primary treatment goals, followed by obstructive symptoms (22%), physical appearance (4%), and ability to see (1%). More than half of the patients chose either pain control (34%) or wound care (27%) as their primary treatment goal, followed by improvement in obstructive symptoms (24%) and in physical appearance (4%).

In the blinded period, 19% (11 out of 57) of CDDP/epi gel treated patients (stratum I, 23%; stratum II, 15%) achieved patient benefit *vs* 9% (three out of 35) of placebo recipients (stratum I, 12%; stratum II, 6%). Although these differences were not significant (overall *P*=0.24, CMH test; both *P*⩾0.46, Fisher's exact test, for strata), active gel recipients were 1.9 to 2.5 times more likely to achieve Patient Benefit than placebo-treated patients. Among benefiters, 64% (nine out of 14) attained their own palliative goal, 57% (eight out of 14) attained the investigator-specified palliative goal, and 29% (four out of 14) attained the investigator-specified preventive goal.

Patient benefit was positively and significantly (*P*=0.024, CMH test) associated with target tumour response in the blinded period among CDDP/epi gel recipients: 43% (six out of 14) of target tumour-responders had patient benefit, whereas only 12% (five out of 43) of non-responders benefited. Most of this difference was in stratum I, in which 50% (five out of 10) of responders benefited *vs* 10% (two out of 21) of non-responders. In seven patients both target tumour response and patient benefit were achieved; in eight tumour response was achieved but not patient benefit; and in seven no target tumour response occurred but patient benefit was still achieved. Changes from baseline in FACT-H&N scores were similar between the groups; FACT-H&N score was not related to target tumour response, patient benefit, or KPS (which was stable during the blinded period in both study groups).

### Drug exposure

During the blinded phase, CDDP/epi gel recipients had a median of four (range one–six) treatments and placebo recipients received a median of three (range one–six) treatments. Median dose per target tumour treatment was 1.6 ml (range, 0.2–8.5) for active gel and 1.7 ml (range, 0.5–10) for placebo, and median cumulative dose was 5.4 ml (range, 1.0–46) and 5.3 ml (range, 0.8–29), respectively. Over 80% of assigned dose was delivered in most treatments; per cent dose delivery ranged from 41 to 213% for active treatment and from 58 to 693% for placebo. Non-delivery of the full assigned dose most commonly occurred because the target tumour could not accommodate the entire dose, in 11% (28 out of 250) of active gel treatments and 10% (11 out of 107) placebo gel treatments.

### Safety

Adverse events during or ⩽20 min after injection were mostly injection site pain, in eight (14%) of CDDP/epi gel recipients (severe in two, moderate in five) and one (3%) of the placebo recipients. Mild to moderate tachycardia in three active gel recipients may have been a reaction to either to pain or to epinephrine released from the injection site.

Local cytotoxic effects (erosion, erythema, eschar, necrosis, swelling, and ulceration) were more frequent and marked in the CDDP/epi gel group than in placebo recipients (Table 3

**Table 3:**
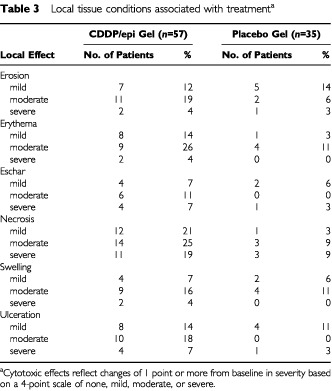
Local tissue conditions associated with treatment^a^

). The development of these effects varied among patients, but generally erythema, swelling, and ulceration were most severe after the first treatment; erosion, necrosis, and eschar were greater following the second treatment; and the effects gradually resolved over the following 2 to 5 months. Responding target tumours, as opposed to non-responding target tumours, tended to develop more erosion (47 *vs* 32%), necrosis (87 *vs* 47%), and eschar (33 *vs* 18%), although these differences were not statistically significant (borderline significance, CMH exact test *P*=0.055, was found for eschar). Aside from local cytotoxic effects, the most common local adverse event sustained beyond the immediate injection period was pain, reported by eight (14%) in the active group (severe in two, moderate in six) and three (9%) in the placebo group (severe in one, moderate in two).

Systemic adverse events of pain, headache, nausea, vomiting, and hypomagnesemia were slightly higher in CDDP/epi gel recipients than placebo recipients (Table 4

**Table 4:**
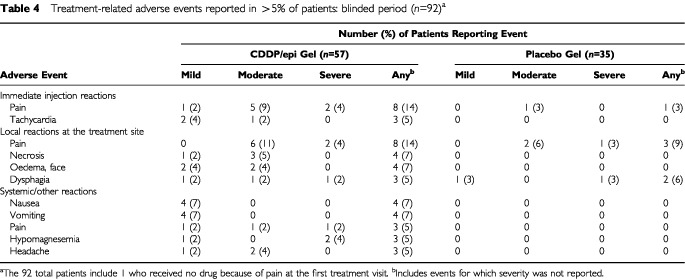
Treatment-related adverse events reported in >5% of patients: blinded period (*n*=92)^a^

). Forty-eight deaths occurred after the start of treatment (26 active, 22 placebo), nearly all due to progression or complications of cancer, and none were judged to be related to treatment. Eight patients assigned to CDDP/epi gel each reported one serious adverse event at least possibly related to treatment: anaemia, allergic reaction, haemorrhage, pallor, blindness, cardiac arrest (non-fatal), oedema, and swelling. Treatment was discontinued following the first five of these events. The allergic reaction was severe, acute, and not related to the collagen component of CDDP/epi gel (anticollagen IgG testing was negative), but rather to another component (possibly cisplatin). The haemorrhage involved acute tumour bleeding at the base of the tongue from a major blood vessel following study drug overdose (⩾125% of assigned study drug volume without reflux) and was managed by cauterization. The (hemifacial) pallor may have been caused by vasospasm secondary to intra-arterial leakage of study medication from the submandibular tumour. Sudden onset of complete right-eye blindness following study drug overdose was probably caused by an isolated right optic nerve lesion (the patient recovered only light sensitivity). Changes in haematology and chemistry values were infrequent and typical of those in patients with advanced cancer. There was no evidence of the renal toxicity or myelotoxicity typical of systemic cisplatin, except for one occurrence of treatment-related anemia (moderate) and one of mild leukopenia.

## DISCUSSION

The results of therapy were assessed by evaluating both local tumour response and patient benefit, a validated measure of how tumour response affected patient well-being. In these heavily pre-treated patients with advanced head and neck squamous cell carcinoma, during the blinded phase, the target tumour responded in 25% (14 out of 57) treated with CDDP/epi gel *vs* 3% (one out of 35) treated with placebo (*P*=0.007). Nearly all responding target tumours (86%) remained in local remission when patients left the study, and the extent of response in patients with PR was notable: median maximal tumour shrinkage of 94% (range, 60–99). The rate of patient benefit in the CDDP/epi gel group (19%) during the blinded phase was more than twice that in the placebo gel group (9%), but the difference was not statistically significant. Patient benefit was positively and significantly (*P*=0.024) associated with target tumour response, supporting the hypothesis that regression of the most clinically dominant tumour can relieve or prevent the patient's most life-impacting symptom.

Target tumour response occurred after a median of 53 days and 4.5 treatments in the 14 actively treated patients who responded during the blinded phase. Responses were durable, lasting a median 64 days, considering the advanced disease and limited expected survival of the study population. Duration of response was a conservative estimate, as local responses continued beyond the last study observation point, but may have impacted patient well-being, as reflected in the patient benefit scores. Mean time to target tumour progression was nearly three times as long in the CDDP/epi gel group as in the placebo group (128 *vs* 44 days).

In this vulnerable study population, CDDP/epi gel had a tolerable safety profile, with adverse effects usually occurring at predictable times (during and shortly after the injection procedure and during the healing process) and involving predictable events (commonly pain and cytotoxicity) generally limited to the tumour site. As expected, the cytotoxic mode of action of CDDP/epi gel resulted in more frequent and more severe cytotoxic events in the active group, which peaked after the second or third treatment and generally resolved during the next 2 to 5 months. Cytotoxic events also occurred in the placebo group, albeit less frequently and probably as a result of advancing cancer (Dvorak, 1986). Systemic adverse effects were infrequent, and the nephrotoxicity, ototoxicity, and myelosuppression usually seen with systemic cisplatin were absent except for treatment-related moderate anaemia and mild leukopenia in one patient each.

The most common adverse effect of treatment was local pain, affecting up to 28% of patients. Although type of analgesia (narcotic *vs* non-narcotic) was not related to occurrence of moderate or severe pain, specific patient characteristics (such as prior regional pain status, general pain tolerance, limitations of comorbidity on pain therapy, and treatment history) may have affected the physician's choice of analgesia.

The next most frequent adverse events among CDDP/epi gel recipients – necrosis (7%), facial oedema (7%), tachycardia (5%), and dysphagia (5%) – were much less common than pain and usually mild or moderate in severity. Nausea and vomiting each were reported in 7% of active gel recipients, and may have been related to pain medication or the pain of the procedure in some cases. Local infection was relatively rare (three cases) and was managed on an outpatient basis by parenteral or oral antibiotics.

The Treatment Goals Questionnaire and patient benefit algorithm demonstrated the clinical benefit of CDDP/epi gel, as indicated by the statistically significant association between target tumour response and patient benefit. In 15 cases, the patient had either target tumour response or patient benefit but not both. In four of these patients, stable disease or tumour regression of <50% (below the level required for tumour response) may have been sufficient to produce patient benefit, and in one patient, benefit preceded documented tumour regression of >50%. In six of the eight cases in which patients achieved target tumour response but not patient benefit, there was no clear evidence that the goals chosen were unrealistic or could not have been reasonably expected as a result of tumour response. In contrast to the Treatment Goals Questionnaire, both the KPS and FACT-H&N were relatively insensitive to the effect of local therapy.

The patients in this study, in which the most problematic tumours were selectively treated, had substantial tumour response rates to CDDP/epi gel even compared with patients with more favourable prognoses (Huber *et al*, 1995), and these responses were often accompanied by life-enhancing clinical benefits. Two-thirds of the responses (nine out of 14) were complete, a CR : PR ratio of about 2 : 1, reversing the typical CR : PR ratio in studies of systemic cisplatin therapy (generally about 1 : 4; Forastiere *et al*, 1992; Jacobs *et al*, 1992; Clavel *et al*, 1994). Therapy with CDDP/epi gel can be administered on an out-patient basis, which is especially important to patients whose remaining life expectancy is limited and needed for family and personal affairs. Out-patient treatment also can protect these frail patients from nosocomial infection and other hospitalisation-associated hazards. The benefits of CDDP/epi gel treatment demonstrated in this study would be difficult to obtain from other treatments available to this patient population, who might not tolerate or might be resistant to systemic chemotherapy, might already have received maximal doses of radiation, and would likely consider the morbidity of additional surgery unacceptable.
